# A novel *VSX1* mutation identified in an individual with keratoconus in India

**Published:** 2009-11-28

**Authors:** Preeti Paliwal, Anuradha Singh, Radhika Tandon, Jeevan S Titiyal, Arundhati Sharma

**Affiliations:** 1 Laboratory of Cyto-Molecular Genetics, Department of Anatomy, All India Institute of Medical Sciences, New Delhi, India; 2 Dr. Rajendra Prasad Centre for Ophthalmic Sciences, All India Institute of Medical Sciences, New Delhi, India

## Abstract

**Purpose:**

To evaluate the possible role of the *VSX1* gene in a group of patients from the Indian subcontinent with keratoconus.

**Methods:**

Molecular analysis of 66 patients with a diagnosis of keratoconus, based on clinical examination and corneal topography, was carried out. DNA extraction from peripheral blood followed by Polymerase Chain Reaction (PCR) amplification of the *VSX1* gene was performed. The entire coding region and the exon–intron junctions of the *VSX1* gene were analyzed by direct sequencing.

**Results:**

A novel change at c.525G>C, replacing amino acid glutamine at position 175 with histidine, was found in one affected individual. One of the previously reported SNPs (rs12480307) was found with equal frequency in both patients and controls.

**Conclusions:**

This is the first report from the Indian subcontinent exploring the role of *VSX1* in the causation of keratoconus. One novel mutation (Q175H) predicted to be a potentially damaging change was seen in an affected individual; this substantiates the importance of this gene but its precise role in disease causation needs further investigation.

## Introduction

Keratoconus is a bilateral, noninflammatory, gradually progressive corneal disorder characterized by progressive thinning and steepening of the central cornea. It usually appears during teenage years, the common symptoms being myopia and astigmatism, and it is one of the major indications for corneal transplantation [1,2].

Keratoconus normally occurs as a sporadic disorder, but both genetic and environmental factors seem to play a role in its causation [3]. A strong family history has been documented in about 6%–10% of patients with both autosomal and X-linked mode of inheritance [4,5]. Its prevalence in first-degree relatives is 13.5% [6] which is between 15 and 64 times higher than for the general population [7]. The etiology of keratoconus is unknown. It arises as an isolated defect but is also known to be associated with conditions such as Ehler–Danlos, Marfan, Apert, Noonan, and Downs Syndrome.

Genome-wide linkage analyses have identified several chromosomal loci and genes that may be associated with keratoconus [8-12]; however, some were eventually excluded [13,14] while for others a conclusive association with the disease is yet to be established. Mutations in *AIPL1*, *CRB1*, and *CRX* have been implicated in patients with Leber congenital amaurosis, rendering them susceptible to keratoconus [15,16].

One of the strongly implicated candidate genes for keratoconus is Visual System Homeobox 1 (*VSX1*) localized to 20p11-q11. Initially this gene was chosen for screening mutations exploring the association between posterior polymorphous corneal dystrophy (PPCD) and keratoconus [12]. *VSX1* is a developmental gene considered important in ocular development and is normally expressed in the developing cornea. *VSX1* mRNA has been found in the outer tier of the inner nuclear layer of the human retina, embryonic craniofacial tissue and the cornea [17]. The gene has 5 exons spanning 6.2 kb of coding sequence [12]. Several genetic variants of the *VSX1* gene [12,18–23] have been reported from various parts of the world but a definite pathogenic role of the genetic variants in causation of keratoconus is not yet established.

The* VSXI* variants include D144E, G160D, P247R, L159M, R166W, H244R, L17P, G160V and N151S (Table 1) which were initially reported as pathogenic but whose pathogenicity could not be confirmed because segregation of these variants was seen in unaffected individuals also. He´on et al. [12] identified a compound heterozygous change with P247R and G160D and reported G160D to be pathogenic and P247R to be nonpathogenic. Another study [18] reported just the opposite, where P247R was found to be co-segregating with keratoconus. The D144E mutation was reported as pathogenic [18,22] in earlier studies but subsequent studies identified its presence in unaffected individuals and suggested this to be a polymorphism [19,20]. The variants R166W, H244R and L159M have been identified in keratoconus patients but these changes did not segregate with the disease phenotype in their family members and hence were not considered sufficiently significant to support a pathogenic role in keratoconus [21]. Similarly, variants G160V and N151S have been identified in patients from the Korean population [23] but these changes have not been reported from other populations.

**Table 1:** Summary of the sequence variants identified in various studies to date.

**Variant number**	**Variant seen**	**Pathogenic /non pathogenic**	**Total number of patients studied**	**Mutation seen in individuals / families**	**Source**
1	L17P	Pathogenic	80	5 /3	[18]
2	D144E	Pathogenic	80	6/2	[18]
		Pathogenic		Ashkenazi Jewish family	[22]
		No familial segregation	85 F	4 (2 [A]; 2 [UA]) /1	[20]
		Non pathogenic	100	1/1	[19]
		Pathogenic/Non pathogenic???	63Kc+90G	2/1	[12]
3	L159 M	Pathogenic(not conserved)	63Kc+90G	4/1	[12]
		Non pathogenic	521	6 (3 [A] ; 2 [UA]; 1 [C]/-	[21]
4	N151S	Pathogenic	249	1/1	[23]
5	G160D	Non pathogenic	80	4/2	[18]
		Identified in PPD not in Keratoconus & not conserved	63	5/1	[12]
6	G160V	Pathogenic	249	13/-	[23]
7	R166W	Pathogenic	63Kc+90G	1/1	[12]
		Non pathogenic	521	NVI	[21]
8	H244R	Pathogenic/non pathogenic??(seen in controls)	63Kc+90G	3/1	[12]
		Non pathogenic	521	3 (2[A], 1[UA])/-	[21]
9	P247R	Pathogenic	80	4 /1	[18]
		Non pathogenic	63Kc+90G	2/1	[12]
		Identified in controls/ non pathogenic	85 F	1/1 [C]	[20]

Table 1 shows the sequence variants identified by various authors and the conclusions reached regarding their pathogenecity. ‘F’ represents families, ‘Kc’ represents patients with keratoconus, ‘G’ represents patients with glaucoma,’ A’ represents variants identified in patients affected with keratoconus, ‘UA’ represents variants identified in unaffected relatives of keratoconus patients, ‘C’ represents variants identified in controls and ‘NVI’ represents no variants identified.

The present study was undertaken in order to search for genetic variations of the *VSX1* gene in the Indian population and to explore its role in causation of keratoconus. Mutational analysis of *VSX1* was carried out in 66 unrelated Indian patients affected with keratoconus in comparison to 100 controls.

## Methods

Patients affected with keratoconus seen in the Cornea and Refractive Surgeries Services and Contact Lens Clinic at Dr. Rajendra Prasad Centre for Ophthalmic Sciences, All India Institute of Medical Sciences, during the period of March 2007 to 2008 were included in the study. The study adhered to the tenets of the Declaration of Helsinki and was approved by the Institutional Ethics Committee. Informed consent was taken from all the patients before being enrolled for the present study. Detailed family histories up to three generations were taken and pedigree charts were constructed. History of ocular or other hereditary disorders was recorded. The existence of consanguinity and the regional birthplace of patients were also noted.

A total of 66 unrelated patients with keratoconus were recruited in the present study: 23 females and 43 males, with 41 patients (62%) aged between 10 and 20. The patients presented with deterioration of vision and were diagnosed based on clinical features, such as stromal thinning, Vogt’s striae, Fleischer’s ring, Munson’s sign, and corneal topography. Topographical features, such as corneal power (K), inferior–superior dioptric asymmetry (I-S), astigmatism (Ast), and skewed radial axis (SRAX) were used to calculate KISA% (a single index that quantifies the irregularity of corneal shape and presence of astigmatism, typical of keratoconus, with good clinical correlation). A total of 100 healthy volunteers with no ocular or other disorders matched for age and gender formed the controls. A peripheral blood sample (5 ml) was collected from all the patients and control subjects after taking informed consent and explaining the nature and possible consequences of study participation.

### DNA extraction and PCR amplification

Genomic DNA was extracted from peripheral blood leukocytes using standard protocols. All the five exons and intron/exon boundaries of *VSX1* were amplified using custom-synthesized oligonucleotide primers as described previously [12]. Each reaction was carried out in a 25 μl mixture containing 2.5 μl 10X PCR buffer with 3.5 mM MgCl_2_, 2.5 mM dNTPs, 10 pM of each primer, 0.7 U Taq DNA polymerase (Roche) and 100 ng genomic DNA. Thermal cycling was performed in a thermal cycler (Applied Biosystem 9700) as described: initial denaturation for 12 min at 95 °C; 35 cycles at 94 °C for 30 s, 62 °C (exons 1–4) or 60 °C (exon 5) for 30 s, 72 °C for 30 s, and a final extension for 10 min at 72 °C.

### DNA Sequencing

All the amplified products were sequenced bidirectionally using BigDye Terminator Mix version 3.1 (Applied Biosystems [ABI], Foster City, CA) according to the manufacturer’s instructions and were analyzed on an ABI-3100 Genetic Analyzer (ABI). Nucleotide sequences were compared with the published *VSX1* cDNA sequence (GenBank NM_014588).

### SIFT and PolyPhen analysis

The potential impact of the amino acid change was assessed with the SIFT analysis tool (Sorting Intolerant From Tolerant) and PolyPhen analysis. These tools produce a multiple sequence alignment from different species of a gene to assess the positions of conserved amino acids and analyze the effect of missense changes on the conserved structure of proteins. The SIFT tool assigns a score to the mutations and a score of <0.05 is considered potentially damaging. PolyPhen analysis uses PSIC software (Position-Specific Independent Counts) to calculate profile scores (PSIC scores) that are logarithmic ratios of the likelihood of a given amino acid occurring at a particular position to the likelihood of this amino acid occurring at any position (background frequency).

## Results

**Figure 1:**
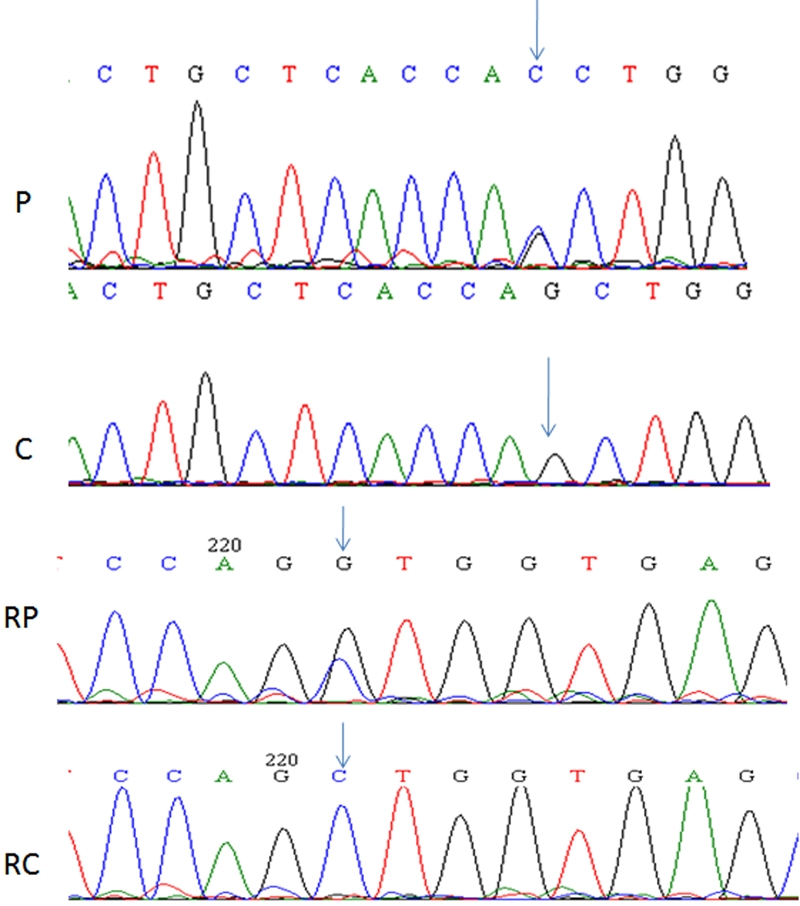
Partial nucleotide sequence of *VSX1* exon 3*. *Partial exon 3 nucleotide sequence of *VSX1* showing G→C transition in the mutated allele of the patient (P) in comparison to control (C). RP represents a partial chromatogram of the reverse nucleotide sequence of the patient in comparison to control (RC). The arrow-head shows the position of the change in the given sequences.

**Figure 2:**
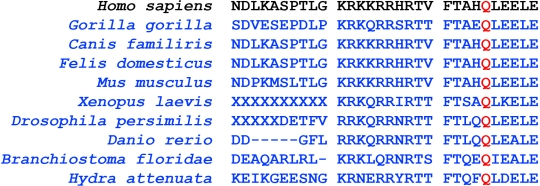
Multiple sequence alignment of the amino acid sequences of VSX1 in different species. The highlighted amino acid glutamine (Q) shown in red at position 175 is conserved in all the orthologs.

The mean age at onset of symptoms among the patients was 18 years. Consanguinity was not present in any of the cases recruited for the present study. The patients were analyzed for the presence of mutations in all the five exons of *VSX1*. Among the patients under study a novel heterozygous nucleotide change c.525G>C (Q175H; GenBank GU138372; ACZ01961) was identified in one individual. The presence of this change was confirmed by a repeat bidirectional sequencing (Figure 1). No similar change was found in any of the 100 normal controls studied.  SIFT tool analysis revealed a score of <0.05 and PolyPhen analysis gave a PSIC score difference of 1.896; both tools predicted that the replaced amino acid would be potentially damaging and would not be tolerated. The amino acid glutamine at position 175 is important and has been conserved throughout the orthologs (Figure 2). A previously reported SNP rs12480307 was also identified in four patients and six controls, segregating with equal frequency in both groups.

## Discussion

In the present study we identified a novel heterozygous change c.525G>C (Q175H; GenBank GU138372; ACZ01961) in an affected individual. SIFT tool and PolyPhen analyses predicted that the mutation Q175H (replacing amino acid glutamine with histidine) could be damaging. The absence of this change in 100 controls is suggestive of it being pathogenic. *VSX1* has previously been implicated in causation of keratoconus according to earlier reports; these established that keratoconus and posterior polymorphic corneal dystrophy are co-localized within the chromosome 20p11-q11 region [12] and identified four sequence variants, two of which were considered pathogenic. To date, several genetic variants of the *VSX1* gene have been reported (Table 1) but their pathogenicity could not always be confirmed because segregation of these variants was also seen in some unaffected individuals. Q175H is a novel mutation, identified in the present study, adding to the database of mutations in the *VSX1* gene and supporting its role in keratoconus. However, its presence in just 1.6% of the patients proves that this may be a minor gene and its exact role during development needs to be documented.

*VSX1* expression is detected in adult retinal and corneal tissue and in embryonic craniofacial tissue [24,25]. The gene is a member of the CVC domain containing a paired-like class of homeodomains (HD) which play a role in craniofacial and ocular development [26]. The homeodomain region folds into three alpha helices; the latter two bind DNA in the major groove of the double helix and the third is the recognition helix that is responsible for amino acids making contact with the DNA bases. The Q175H change is present in the HD region of *VSX1*, where a neutral amino acid is replaced by a polar one. This may affect the binding of *VSX1* protein with the DNA and modify the transcription rate. The onset for keratoconus is typically seen around the teenage years and so its role in normal eye development cannot be ruled out. In the light of previous studies, there is still insufficient evidence for the pathogenic role of *VSX1* alone in causation of keratoconus. However, in our study potentially damaging mutation was detected, although only in 1.6% of the patients. Recently, *CRB1* mutations were found to be associated with keratoconus in patients with Leber Congenital Amaurosis; in this case  the authors suggested that the *CRB1* mutations could make patients more susceptible to developing keratoconus [15]. Therefore, the *VSX1* gene might have a small effect by itself, yet operate in conjunction with other genes or environmental factors in causation of keratoconus.

To conclude, to the best of our knowledge this is the first report from the Indian subcontinent exploring the causative role of the *VSX1* gene in keratoconus. We have identified one novel mutation, Q175H, predicted to cause a pathogenic change which would not be tolerated. This study adds one novel pathogenic mutation to the existing repertoire of *VSX1* mutations but its wider role still needs to be explored.
